# Spacecraft Attitude Measurement and Control Using VSMSCSG and Fractional-Order Zeroing Neural Network Adaptive Steering Law

**DOI:** 10.3390/s24030766

**Published:** 2024-01-24

**Authors:** Lei Li, Yuan Ren, Weijie Wang, Weikun Pang

**Affiliations:** 1 Graduate School, Space Engineering University, Beijing 101400, China; 14291011@bjtu.edu.cn; 2 Department of Basic Course, Space Engineering University, Beijing 101400, China; 3 Department of Astronautical Science and Technology, Space Engineering University, Beijing 101400, China; wangwjie@126.com

**Keywords:** variable speed magnetically suspended control sensitive gyroscope, fractional-order zeroing neural network, steering law, spacecraft attitude measurement and control integration

## Abstract

In order to improve the accuracy and convergence speed of the steering law under the conditions of high dynamics, high bandwidth, and a small deflection angle, and in an effort to improve attitude measurement and control accuracy of the spacecraft, a spacecraft attitude measurement and control method based on variable speed magnetically suspended control sensitive gyroscopes (VSMSCSGs) and the fractional-order zeroing neural network (FO-ZNN) steering law is proposed. First, a VSMSCSG configuration is designed to realize attitude measurement and control integration in which the VSMSCSGs are employed as both actuators and attitude-rate sensors. Second, a novel adaptive steering law using FO-ZNN is designed. The matrix pseudoinverses are replaced by FO-ZNN outputs, which solves the problem of accuracy degradation in the traditional pseudoinverse steering laws due to the complexity of matrix pseudoinverse operations under high dynamics conditions. In addition, the convergence and robustness of the FO-ZNN are proven. The results show that the proposed FO-ZNN converges faster than the traditional zeroing neural network under external disturbances. Finally, a new weighting function containing rotor deflection angles is added to the steering law to ensure that the saturation of the rotor deflection angles can be avoided. Semi-physical simulation results demonstrate the correctness and superiority of the proposed method.

## 1. Introduction

Spacecraft attitude maneuvering and measurement are affected by micro-vibrations in space, and vibration suppression becomes more difficult when the bandwidth of vibration is high [1,2]. A VSMSCSG [3] is a novel inertial device for spacecraft attitude control and measurement. The rotor of the VSMSCSG is driven by a Lorentz force magnetic bearing (LFMB) [4], enabling the VSMSCSG to simultaneously measure and control spacecraft attitude in a suitable configuration. The rotor has a two-degree-of-freedom (DOF) micro-gimbal moment output capability; the bandwidth of the moment can be up to 100 Hz, which has great advantages in terms of spacecraft micro-vibration suppression. In addition, the VSMSCSG has a two-degree-of-freedom attitude measurement capability. In order to coordinate the control of each VSMSCSG in a given configuration so that the configuration outputs the desired moment for attitude control, it is necessary to design a high-performance steering law for the VSMSCSG configuration under high bandwidth conditions.

Li [5] proposed an integrated method of spacecraft attitude measurement and control using a VSMSCSG configuration; however, since this configuration contained only four VSMSCSGs, the attitude measurement accuracy was limited, and the computation was complex. In addition, the study did not involve the design of the steering laws, which affects the accuracy of the obtained attitude control.

Existing steering laws can be categorized into frame angle steering laws, frame angular velocity steering laws, and frame angular acceleration steering laws. The frame angular velocity steering laws are the most intensively studied, such as the Penrose–Moore pseudoinverse steering laws [6,7], the pseudoinverse steering law with zero motion [8], the gradient-type steering law [9], and the singular robust inverse steering law [10].

Xia [11] presented a steering law for a pyramidal single gimbal control moment gyro system for spacecraft attitude control. Wang [12] proposed a new strategy for solving the singularity problem, which reveals the structural properties in terms of the upper and lower bounds of the flywheel governing speed. Xia [13] proposed an adaptive nonlinear pseudoinverse reversal law to effectively suppress the effect of rotor tilt saturation on the sensitive gyro cluster [14]. Zhang [15] designed an anti-saturation steering law based on the pseudoinverse; he added a weight parameter to the steering law to avoid the angle saturation.

The above steering laws solve the pseudoinverse of the Jacobian matrix to obtain the frame angular velocity, which is simple and easy to implement. However, they inevitably involve pseudoinverse solutions of time-varying matrices, which are more complicated to solve due to the high bandwidth of the VSMSCSG at output motion and because its Jacobian matrix consists of a series of small quantities with highly time-varying bandwidths.

Some research focuses on solving the steering law question with neural networks. Zhong [16] proposed a steering law using a radial basis function (RBF) neural network which enables the single gimbal control moment gyroscopes to avoid singularity. Wei [17] designed a recurrent neural network to reduce the error due to the pseudoinverse operation in the steering law. However, when the rotor deflection bandwidth is high, the moment error caused by rotational speed deviation, sensor error, mounting error, etc. is difficult to avoid. In addition, since the deflection angle of a VSMSCSG is limited to ±2° [18], traditional steering laws inevitably cause the saturation of the rotor deflection angle, resulting in output moment limitation. Existing steering laws have limitations in solving these problems.

Some studies focus on tackling the time-varying matrix pseudoinversion problem with zeroing neural networks [19,20]. Hu [21] designed two zeroing neural networks to solve the inverse problem of time-varying matrices. He improved the activation function of the ZNN and proved that the activation function can accelerate the convergence of the ZNN while also improving the robustness of the network. Lin [22] proposed two novel nonlinearly activated recurrent neural networks with finite-time convergence. Predrag [23] proposed an integration-enhanced noise-tolerant zeroing neural network to approximate time-varying outer inverse with a prescribed range and null space. Si [24] proposed a finite-time convergent nonconvex zeroing neural network model with a faster convergence rate than the original zeroing neural network model.

These studies show that the zeroing neural network has outstanding advantages in solving time-varying matrix pseudoinversion problems. However, these ZNNs do not address the issues of convergence accuracy and speed under high bandwidth conditions and cannot be directly applied to VSMSCSG steering law design [25]. When used in VSMSCSG steering law, zeroing neural networks need to be optimally designed. Fractional-order differentiation is widely used in control applications due to its fast convergence and memory characteristics [26,27], which provide a feasible way to improve the convergence performance of zeroing neural networks.

In order to solve the above problems, a fractional-order zeroing neural network steering law is proposed in this paper. A VSMSCSG configuration is designed to realize attitude measurement and control simultaneously. Compared to [5], the attitude measurement accuracy is improved and the computation is simplified due to the additional VSMSCSG in the configuration. A fractional-order activation function is designed for the first time to accelerate the convergence speed of the network. The convergence and robustness of the proposed network are proven. An adaptive zeroing neural network steering law is developed to replace the matrix pseudoinverse with the output of the FO-ZNN. Based on this, a weighting matrix with a nonlinear function is added to the steering law to ensure that saturation can be avoided by de-deflecting the other degrees of freedom when the angle is close to saturation. The semi-physical simulation results show that the proposed zeroing neural network steering law has good performance in spacecraft attitude control.

## 2. Attitude Measurement and Control Method Using VSMSCSG Configuration

### 2.1. The Structure and Principle of the VSMSCSG

**Figure 1:**
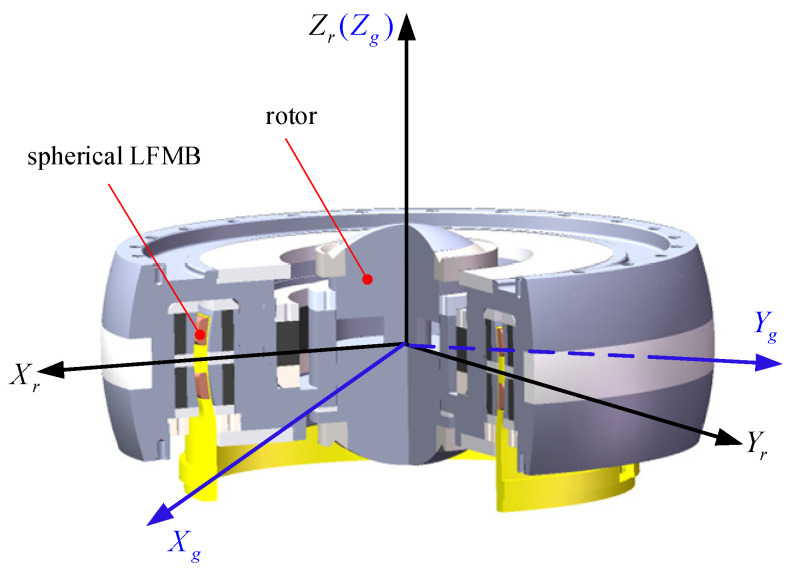
VSMSCSG rotor structure Schematic diagram.

The VSMSCSG structure is shown in Figure 1, where *OX_g_Y_g_Z_g_* is the LFMB coordinate system and is connected to the LFMB stator, *O* is the centroid of the rotor, and *OX_r_Y_r_Z_r_* is the rotor coordinate system and is tied up with the rotor. The rotor deflects at an angle of *α* around *OX_g_* and an angle of *β* around *OY_g_*. The synthetic deflection of the rotor is around *OX_r_*. Ω is the rotor speed.

The relationship between the control moment $M_{g}$ output by LFMB and the rotor deflection angles can be expressed as [5]

(1) $$M=\left(\begin{array}{c} M_{x}\\ M_{y} \end{array}\right)=\left(\begin{array}{c} J_{r}\ddot{\alpha }+J_{z}\Omega \dot{\beta }\\ J_{r}\ddot{\beta }-J_{z}\Omega \dot{\alpha } \end{array}\right)=\left(\begin{array}{c} 4NBLl_{m}I_{\alpha }\\ -4NBLl_{m}I_{\beta } \end{array}\right)$$

where ***M****_x_* and ***M****_y_* represent the moments along *X_g_*- and *Y_g_*-axes, respectively; *J_r_* and *J_z_* are the rotor radial inertia moment and axial inertia moment, respectively; *N* represents the number of turns of the LFMB coils; *B* represents the magnetic density at the coil; *L* represents the coil length perpendicular to the magnetic field; *l_m_* represents the radius of LFMB stator; and *I_α_* and *I_β_* represent the control current through the *X_g_*- and *Y_g_*-axis coils of the LFMB, respectively [5].

It can be seen from (1) that the VSMSCSG can output a three-degree-of-freedom control moment by changing the rotor deflection angle and the rotor speed, and it can calculate two-degree-of-freedom angle information by simultaneously detecting the currents in the LFMB coils.

### 2.2. Attitude Measurement and Control Integration Method

**Figure 2:**
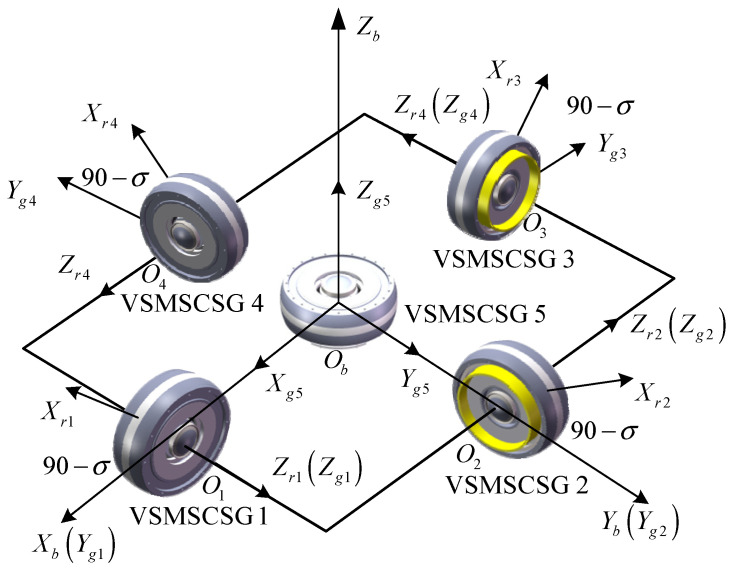
Schematic diagram of VSMSCSG configuration.

The VSMSCSG configuration is shown in Figure 2, where *O_b_X_b_Y_b_Z_b_* is the spacecraft body’s coordinate, and *O_b_X_b_*, *O_b_Y_b_*, and *O_b_Z_b_* are the roll, yaw, and pitch axes, respectively.

The configuration is composed of 5 VSMSCSGs. The rotor of VSMSCSG 5 does not deflect. *O*_1_*Z_g_*_1_, *O*_2_*Z_g_*_2_, *O*_3_*Z_g_*_3_, and *O*_4_*Z_g_*_4_ coincide with *O_b_Y_b_*, −*O_b_X_b_*, −*O_b_Y_b_*, and *O_b_X_b_*, respectively. The angle between the *O*_1_*X_r_*_1_ and *O*_1_*X_g_*_1_, *O*_2_*X_r_*_2_ and *O*_2_*X_g_*_2_, *O*_3_*X_r_*_3_ and *O*_3_*X_g_*_3_, and *O*_4_*X_r_*_4_ and *O*_4_*X_g_*_4_ axes is σ.

The angular momentum of the VSMSCSG configuration in the spacecraft body coordinate system can be expressed as

(2) $$H=\sum_{i=1}^{5}C_{gi}^{b}C_{ri}^{g}h_{i}$$

where ***H*** is the configuration of the angular momentum, $C_{ri}^{g}$ is the transformation matrix from the *i*th rotor coordinate system to the *i*th LFMB coordinate system, **$C_{gi}^{b}$** is the transformation matrix from the *i*th LFMB coordinate system to the spacecraft body’s coordinate system, and ***h_i_*** is the projection of the *i*th rotor angular momentum in the rotor coordinate system [5].

The angular momentum associated with the rotor deflection angular velocity is set as a disturbance. The rotor angular momentum vector is approximately equivalent to

(3) $$h_{i}\approx J_{z}\Omega_{i}\left[\begin{array}{l} \cos\alpha_{i}\sin\beta_{i}\\ -\sin\alpha_{i}\\ \cos\alpha_{i}\cos\beta_{i} \end{array}\right]$$

Since the rotor of VSMSCSG 5 does not deflect, its angular momentum can be expressed as $h_{5}={\left[\begin{array}{ccc} 0 & 0 & J_{z}\Omega_{5} \end{array}\right]}^{T}$. Supposing that $\sigma =45^{\circ }$, the output moment can be expressed in three parts:

(4) $$u_{\alpha }=A\dot{\alpha }$$

(5) $$u_{\beta }=B\dot{\beta }$$

(6) $$u_{\Omega }=C\dot{\Omega }$$

Combining (1)–(3) yields, one has

(7) $$\begin{array}{l} \dot{H}=\left[\begin{array}{c} -J_{z}\Omega_{1}\cos\alpha_{1}\;\;\;J_{z}\Omega_{2}\sin\alpha_{2}\cos\beta_{2}\;\;kJ_{z}\Omega_{3}\cos\alpha_{3}\;\;-J_{z}\Omega_{4}\sin\alpha_{4}\cos\beta_{4}\\ -J_{z}\Omega_{1}\sin\alpha_{1}\cos\beta_{1}\;\;-J_{z}\Omega_{2}\cos\alpha_{2}\;\;J_{z}\Omega_{3}\sin\alpha_{3}\cos\beta_{3}\;\;J_{z}\Omega_{4}\cos\alpha_{4}\\ J_{z}\Omega_{1}\cos\alpha_{1}\;\;\;\;\;\;J_{z}\Omega_{2}\cos\alpha_{2}\;\;\;\;\;\;\;J_{z}\Omega_{3}\cos\alpha_{3}\;\;\;\;\;\;\;J_{z}\Omega_{4}\cos\alpha_{4} \end{array}\right]\left[\begin{array}{c} {\dot{\alpha }}_{1}\\ {\dot{\alpha }}_{2}\\ {\dot{\alpha }}_{3}\\ {\dot{\alpha }}_{4} \end{array}\right]+\left[\begin{array}{c} \begin{array}{l} -J_{z}\Omega_{1}\cos\beta_{1}\cos\alpha_{1}\;J_{z}\Omega_{2}\sin\beta_{2}\cos\alpha_{1}\ldots \\ \ldots J_{z}\Omega_{3}\cos\beta_{3}\cos\alpha_{3}\;-J_{z}\Omega_{4}\sin\beta_{4}\cos\alpha_{4} \end{array}\\ \begin{array}{l} -J_{z}\Omega_{1}\sin\beta_{1}\cos\alpha_{1}\;\;-J_{z}\Omega_{2}\cos\beta_{2}\cos\alpha_{2}\ldots \\ \ldots J_{z}\Omega_{3}\sin\beta_{3}\cos\alpha_{3}\;\;J_{z}\Omega_{4}\cos\beta_{4}\cos\alpha_{4} \end{array}\\ \begin{array}{l} J_{z}\Omega_{1}\cos\beta_{1}\cos\alpha_{1}\;J_{z}\Omega_{2}\cos\beta_{2}\cos\alpha_{2}\ldots \\ \ldots J_{z}\Omega_{3}\cos\beta_{3}\cos\alpha_{3}\;J_{z}\Omega_{4}\cos\beta_{4}\cos\alpha_{4} \end{array} \end{array}\right]\left[\begin{array}{c} {\dot{\beta }}_{1}\\ {\dot{\beta }}_{2}\\ {\dot{\beta }}_{3}\\ {\dot{\beta }}_{4} \end{array}\right]\\ \;\;\;\;\;+\left[\begin{array}{c} \begin{array}{l} J_{z}\cos\alpha_{1}\sin\beta_{1}-J_{z}\sin\alpha_{1}\;\;-J_{z}\cos\alpha_{2}\cos\beta_{2}\ldots \\ \ldots -J_{z}\cos\alpha_{3}\sin\beta_{3}+J_{z}\sin\alpha_{1}\;\;\;J_{z}\cos\alpha_{4}\cos\beta_{4}\;\;0 \end{array}\\ \begin{array}{l} J_{z}\cos\alpha_{1}\cos\beta_{1}\;\;J_{z}\cos\alpha_{2}\sin\beta_{2}-J_{z}\sin\alpha_{2}\ldots \\ \ldots -J_{z}\cos\alpha_{3}\cos\beta_{3}\;\;-J_{z}\cos\alpha_{2}\sin\beta_{2}+J_{z}\sin\alpha_{4}\;\;0 \end{array}\\ \begin{array}{l} J_{z}\cos\alpha_{1}\sin\beta_{1}+J_{z}\sin\alpha_{1}\;\;J_{z}\cos\alpha_{2}\sin\beta_{2}+J_{z}\sin\alpha_{2}\ldots \\ \ldots J_{z}\cos\alpha_{3}\sin\beta_{3}+J_{z}\sin\alpha_{3}\;\;J_{z}\cos\alpha_{4}\sin\beta_{4}+J_{z}\sin\alpha_{4}\;\;J_{z} \end{array} \end{array}\right]\left[\begin{array}{c} {\dot{\Omega }}_{1}\\ {\dot{\Omega }}_{2}\\ {\dot{\Omega }}_{3}\\ \begin{array}{c} {\dot{\Omega }}_{4}\\ {\dot{\Omega }}_{5} \end{array} \end{array}\right] \end{array}$$

where $\Omega_{i}$ is the rotor speed of the *i*th VSMSCSG. It can be obtained from (4)–(7) that

(8) $$A=\left[\begin{array}{c} -J_{z}\Omega_{1}\cos\alpha_{1}\;\;\;J_{z}\Omega_{2}\sin\alpha_{2}\cos\beta_{2}\;\;kJ_{z}\Omega_{3}\cos\alpha_{3}\;\;-J_{z}\Omega_{4}\sin\alpha_{4}\cos\beta_{4}\\ -J_{z}\Omega_{1}\sin\alpha_{1}\cos\beta_{1}\;\;-J_{z}\Omega_{2}\cos\alpha_{2}\;\;J_{z}\Omega_{3}\sin\alpha_{3}\cos\beta_{3}\;\;J_{z}\Omega_{4}\cos\alpha_{4}\\ J_{z}\Omega_{1}\cos\alpha_{1}\;\;\;\;\;\;J_{z}\Omega_{2}\cos\alpha_{2}\;\;\;\;\;\;\;J_{z}\Omega_{3}\cos\alpha_{3}\;\;\;\;\;\;\;J_{z}\Omega_{4}\cos\alpha_{4} \end{array}\right]$$

(9) $$B=\left[\begin{array}{c} \begin{array}{l} -J_{z}\Omega_{1}\cos\beta_{1}\cos\alpha_{1}\;J_{z}\Omega_{2}\sin\beta_{2}\cos\alpha_{1}\ldots \\ \ldots J_{z}\Omega_{3}\cos\beta_{3}\cos\alpha_{3}\;-J_{z}\Omega_{4}\sin\beta_{4}\cos\alpha_{4} \end{array}\\ \begin{array}{l} -J_{z}\Omega_{1}\sin\beta_{1}\cos\alpha_{1}\;\;-J_{z}\Omega_{2}\cos\beta_{2}\cos\alpha_{2}\ldots \\ \ldots J_{z}\Omega_{3}\sin\beta_{3}\cos\alpha_{3}\;\;J_{z}\Omega_{4}\cos\beta_{4}\cos\alpha_{4} \end{array}\\ \begin{array}{l} J_{z}\Omega_{1}\cos\beta_{1}\cos\alpha_{1}\;J_{z}\Omega_{2}\cos\beta_{2}\cos\alpha_{2}\ldots \\ \ldots J_{z}\Omega_{3}\cos\beta_{3}\cos\alpha_{3}\;J_{z}\Omega_{4}\cos\beta_{4}\cos\alpha_{4} \end{array} \end{array}\right]$$

(10) $$C=\left[\begin{array}{c} \begin{array}{l} J_{z}\cos\alpha_{1}\sin\beta_{1}-J_{z}\sin\alpha_{1}\;\;-J_{z}\cos\alpha_{2}\cos\beta_{2}\ldots \\ \ldots -J_{z}\cos\alpha_{3}\sin\beta_{3}+J_{z}\sin\alpha_{1}\;\;\;J_{z}\cos\alpha_{4}\cos\beta_{4}\;\;0 \end{array}\\ \begin{array}{l} J_{z}\cos\alpha_{1}\cos\beta_{1}\;\;J_{z}\cos\alpha_{2}\sin\beta_{2}-J_{z}\sin\alpha_{2}\ldots \\ \ldots -J_{z}\cos\alpha_{3}\cos\beta_{3}\;\;-J_{z}\cos\alpha_{2}\sin\beta_{2}+J_{z}\sin\alpha_{4}\;\;0 \end{array}\\ \begin{array}{l} J_{z}\cos\alpha_{1}\sin\beta_{1}+J_{z}\sin\alpha_{1}\;\;J_{z}\cos\alpha_{2}\sin\beta_{2}+J_{z}\sin\alpha_{2}\ldots \\ \ldots J_{z}\cos\alpha_{3}\sin\beta_{3}+J_{z}\sin\alpha_{3}\;\;J_{z}\cos\alpha_{4}\sin\beta_{4}+J_{z}\sin\alpha_{4}\;\;J_{z} \end{array} \end{array}\right]$$

The three-axis attitude of the spacecraft can be obtained by utilizing the current information from the LFMB coils of VSMSCSG 1~VSMSCSG 5. From (1), as for the VSMSCSG 1~VSMSCSG 4, one has

(11) $$\left\{ \begin{array}{l} M_{1x}=J_{z}\Omega_{1}\left({\dot{\beta }}_{1}+\omega_{x}\right)+\left(J_{z}-J_{r}\right)\omega_{y}\left({\dot{\beta }}_{1}+\omega_{x}\right)+J_{r}\left({\ddot{\alpha }}_{1}+{\dot{\omega }}_{z}\right)\\ M_{1y}=-J_{z}\Omega_{1}\left({\dot{\alpha }}_{1}+\omega_{z}\right)-\left(J_{z}-J_{r}\right)\omega_{y}\left({\dot{\alpha }}_{1}+\omega_{z}\right)+J_{r}\left({\ddot{\beta }}_{1}+{\dot{\omega }}_{x}\right) \end{array}\right.$$

(12) $$\left\{ \begin{array}{l} M_{2x}=J_{z}\Omega_{2}\left({\dot{\beta }}_{2}+\omega_{y}\right)+\left(J_{z}-J_{r}\right)\left(-\omega_{x}\right)\left({\dot{\beta }}_{2}+\omega_{y}\right)+J_{r}\left({\ddot{\alpha }}_{2}+{\dot{\omega }}_{z}\right)\\ M_{2y}=-J_{z}\Omega_{2}\left({\dot{\alpha }}_{2}+\omega_{z}\right)-\left(J_{z}-J_{r}\right)\left(-\omega_{x}\right)\left({\dot{\alpha }}_{2}+\omega_{z}\right)+J_{r}\left({\ddot{\beta }}_{2}+{\dot{\omega }}_{y}\right) \end{array}\right.$$

(13) $$\left\{ \begin{array}{l} M_{3x}=J_{z}\Omega_{3}\left({\dot{\beta }}_{3}-\omega_{x}\right)+\left(J_{z}-J_{r}\right)\left(-\omega_{y}\right)\left({\dot{\beta }}_{3}-\omega_{x}\right)+J_{r}\left({\ddot{\alpha }}_{3}+{\dot{\omega }}_{z}\right)\\ M_{3y}=-J_{z}\Omega_{3}\left({\dot{\alpha }}_{3}+\omega_{z}\right)-\left(J_{z}-J_{r}\right)\left(-\omega_{y}\right)\left({\dot{\alpha }}_{3}+\omega_{z}\right)+J_{r}\left({\ddot{\beta }}_{3}-{\dot{\omega }}_{x}\right) \end{array}\right.$$

(14) $$\left\{ \begin{array}{l} M_{4x}=J_{z}\Omega_{4}\left({\dot{\beta }}_{4}-\omega_{y}\right)+\left(J_{z}-J_{r}\right)\omega_{x}\left({\dot{\beta }}_{4}-\omega_{y}\right)+J_{r}\left({\ddot{\alpha }}_{4}+{\dot{\omega }}_{z}\right)\\ M_{4y}=-J_{z}\Omega_{4}\left({\dot{\alpha }}_{4}+\omega_{z}\right)-\left(J_{z}-J_{r}\right)\omega_{x}\left({\dot{\alpha }}_{4}+\omega_{z}\right)+J_{r}\left({\ddot{\beta }}_{4}-{\dot{\omega }}_{y}\right) \end{array}\right.$$

As for VSMSCSG 5, one has

(15) $$\{\begin{array}{c} M_{5x}=J_{r}{\dot{\omega }}_{x}+J_{z}\Omega \omega_{y}\\ M_{5y}=J_{r}{\dot{\omega }}_{y}-J_{z}\Omega \omega_{x} \end{array}$$

where ***ω*** = [*ω_x_ ω_y_ ω_z_*]^T^ represents the three-axis rotational angular velocity of spacecraft. Combining (10)–(13) yields

(16) $$\begin{array}{l} M_{1x}-M_{3x}=\left(J_{z}-J_{r}\right)\left({\dot{\beta }}_{1}+{\dot{\beta }}_{3}\right)\cdot \omega_{y}+J_{z}\left(\Omega_{1}+\Omega_{3}\right)\cdot \omega_{x}+J_{z}\left({\dot{\beta }}_{1}\Omega_{1}-{\dot{\beta }}_{3}\Omega_{3}\right)+J_{r}\left({\ddot{\alpha }}_{1}-{\ddot{\alpha }}_{3}\right)\\ M_{2x}-M_{4x}=-\left(J_{z}-J_{r}\right)\left({\dot{\beta }}_{2}+{\dot{\beta }}_{4}\right)\cdot \omega_{x}+J_{z}\left(\Omega_{2}+\Omega_{4}\right)\cdot \omega_{y}+J_{z}\left({\dot{\beta }}_{2}\Omega_{2}-{\dot{\beta }}_{4}\Omega_{4}\right)+J_{r}\left({\ddot{\alpha }}_{2}-{\ddot{\alpha }}_{4}\right) \end{array}$$

There are two unknowns and two equations in (15), which means that $\omega_{x}$ and $\omega_{y}$ can be solved. Then, from (10) and (14), one can calculate $\omega_{z}$. Compared to [5], the attitude measurement computation is simplified. Thus, we have realized the integration of three-axis attitude measurement and control for the spacecraft.

The spacecraft dynamics equation can be written as

(17) $$\left(J_{0}+\Delta J\right)\dot{\omega }+\omega \times \left(J_{0}+\Delta J\right)\omega =u+T_{d}$$

where ***J***_0_ represents the rotational inertia matrix of the spacecraft, Δ***J*** represents the unknown disturbance in rotational inertia, and ***T_d_*** represents the external disturbance [5]. Moreover, $u={\left[u_{x}\;u_{y}\;u_{z}\right]}^{T}$ represents the attitude control moment which can be written as

(18) $$u=u_{\alpha }+u_{\beta }+u_{\Omega }=-({\dot{H}}_{b}+\omega \times H_{b})$$

## 3. Design of Fractional-Order Zeroing Neural Network Steering Law

### 3.1. Construction of Fractional-Order Zeroing Neural Network and Analysis of Its Convergence Performance

The traditional zeroing neural network can be expressed as

(19) $$\begin{array}{l} M^{T}\left(t\right)M\left(t\right)\dot{X}\left(t\right)=-\left({\dot{M}}^{T}\left(t\right)M\left(t\right)+M^{T}\left(t\right)\dot{M}\left(t\right)\right)X\left(t\right)\\ +{\dot{M}}^{T}\left(t\right)-F\left(M^{T}\left(t\right)M\left(t\right)X\left(t\right)-M^{T}\left(t\right)\right) \end{array}$$

where $M\left(t\right)\in R^{m\times n}$ is a dynamic full rank matrix and $X\left(t\right)\in R^{n\times m}$ is the the pseudoinverse of M(t) as well as being output of the neural network. $F\left(\cdot \right):R^{n\times m}\to R^{n\times m}$ represents a matrix mapping.

The error matrix E(t) is defined as

(20) $$E\left(t\right)=M^{T}\left(t\right)M\left(t\right)X\left(t\right)-M^{T}\left(t\right)$$

The activation function can be expressed as

(21) $$\frac{dE\left(t\right)}{dt}=-F\left(E\left(t\right)\right)$$

It has been proven that when F(E(t)) is a monotone singular function, the output of the zeroing neural network can approach the pseudoinverse solution [28].

A feasible high-performance finite-time activation function (FTAF) can be expressed as

(22) $$F\left(E\left(t\right)\right)=-\beta_{1}{\mathrm{sgn}}^{v}\left(E\left(t\right)\right)-\beta_{2}E\left(t\right)$$

where $\beta_{1}>0\;,\beta_{2}>0\;,v\in \left(0,1\right)$.

In this paper, a fractional-order zeroing neural network is proposed to improve the approximation speed and robustness of the network.

The fractional-order finite-time activation function (FOFTAF) is designed as follows:

(23) $$\frac{dE}{dt}=-\beta_{1}D^{r}{\mathrm{sgn}}^{v}\left(E\left(t\right)\right)-\beta_{2}E\left(t\right)$$

Thus, the fractional-order zeroing neural network can be expressed as

(24) $$\begin{array}{l} M^{T}\left(t\right)M\left(t\right)\dot{X}\left(t\right)=-\left({\dot{M}}^{T}\left(t\right)M\left(t\right)+M^{T}\left(t\right)\dot{M}\left(t\right)\right)X\left(t\right)\\ +{\dot{M}}^{T}\left(t\right)-\beta_{1}D^{r}{\mathrm{sgn}}^{v}\left(F\left(M^{T}\left(t\right)M\left(t\right)X\left(t\right)-M^{T}\left(t\right)\right)\right)\\ -\beta_{2}\left(F\left(M^{T}\left(t\right)M\left(t\right)X\left(t\right)-M^{T}\left(t\right)\right)\right) \end{array}$$

**Theorem** **1.** *The fractional-order zeroing neural network is asymptotically stable, its output will converge to the theoretical pseudoinverse under bounded disturbance, and the network error is bounded.*

**Proof.**  According to the definition of RL fractional-order differentiation

(25) Dtr0RLf(t)=1Γ(r)∫0t(t−τ)r−1f(τ)dτ

where *r* is the differential order satisfying r∈(0,1). One has

(26) $$\Gamma \left(r\right)=\int_{0}^{\infty }e^{-t}t^{r-1}dt$$

If v≥−1, one has

(27) Dtr0RLtv=Γ(1+v)Γ(1+v−r)tv−r

Choosing element $e_{ij}\left(t\right)$ of E(t) as the object of study, one has

(28) $$\frac{de_{ij}\left(t\right)}{dt}=-\beta_{1}D^{r}{\mathrm{sgn}}^{v}\left(e_{ij}\left(t\right)\right)-\beta_{2}e_{ij}\left(t\right)$$

where

(29) $${\mathrm{sgn}}^{v}\left(e_{ij}\right)=\{\begin{array}{c} {\left|e_{ij}\right|}^{v}\;\;\;e_{ij}>0\\ 0\;\;\;\;\;\;\;e_{ij}=0\\ -{\left|e_{ij}\right|}^{v}\;\;e_{ij}<0 \end{array}$$

Suppose a bounded disturbance Δ(t) is added to the model:

(30) $$\frac{dE\left(t\right)}{dt}=-\beta_{1}D^{r}{\mathrm{sgn}}^{v}\left(E\left(t\right)\right)-\beta_{2}E\left(t\right)+\Delta \left(t\right)$$

Vectorize the matrix as

(31) $$\dot{e}\left(t\right)=-\beta_{1}D^{r}{\mathrm{sgn}}^{v}\left(e\left(t\right)\right)-\beta_{2}e\left(t\right)+\delta \left(t\right)$$

where $e\left(t\right):=\mathrm{vec}\left(E\left(t\right)\right)\in R^{mn\times 1}$, $\delta \left(t\right):=\mathrm{vec}\left(\Delta \left(t\right)\right)\in R^{mn\times 1}$.

Suppose the following Lyapunov function:

(32) $$V=\frac{1}{2}e^{2}$$

(33) $$\dot{V}=e{\left(t\right)}^{T}\dot{e}\left(t\right)=e{\left(t\right)}^{T}\left(-\beta_{1}D^{r}{\mathrm{sgn}}^{v}\left(e\left(t\right)\right)-\beta_{2}e\left(t\right)+\delta \left(t\right)\right)$$

Since the fractional-order activation function is a monotone odd function, one has

(34) $$\begin{array}{l} \dot{V}=\sum_{i=1}^{mn}e_{i}\left(t\right)\left(-\beta_{1}\frac{\Gamma \left(v+1\right)}{\Gamma \left(v-r+1\right)}e_{i}{\left(t\right)}^{v-r}-\beta_{2}e_{i}\left(t\right)+\delta_{i}\left(t\right)\right)\\ \;\;\;=\sum_{i=1}^{mn}\left(-\beta_{1}\frac{\Gamma \left(v+1\right)}{\Gamma \left(v-r+1\right)}{\left|e_{i}\left(t\right)\right|}^{1+v-r}-\beta_{2}{\left|e_{i}\left(t\right)\right|}^{2}+e_{i}\left(t\right)\delta_{i}\left(t\right)\right)\\ \;\;\;\leq \sum_{i=1}^{mn}\left(-\beta_{1}\frac{\Gamma \left(v+1\right)}{\Gamma \left(v-r+1\right)}{\left|e_{i}\left(t\right)\right|}^{1+v-r}-\beta_{2}{\left|e_{i}\left(t\right)\right|}^{2}+e_{i}\left(t\right)\left|\delta \left(t\right)\right|\right) \end{array}$$

Suppose that

(35) $$g\left(e_{i}\left(t\right)\right)=\sum_{i=1}^{mn}\left(-\beta_{1}\frac{\Gamma \left(v+1\right)}{\Gamma \left(v-r+1\right)}{\left|e_{i}\left(t\right)\right|}^{1+v-r}-\beta_{2}{\left|e_{i}\left(t\right)\right|}^{2}\right)$$

(36) $$h\left(e_{i}\right)=\sum_{i=1}^{mn}\left(e_{i}\left|\delta \right|\right)$$

If $\left|e_{i}\left(t\right)\right|=0$ holds for ∀i∈{1,2,…,mn}, one has $\dot{V}\leq 0$, which means the fractional-order zeroing neural network is asymptotically stable and its output will converge to the theoretical pseudoinverse under bounded disturbance.

If there exists $\left|e_{i}\left(t\right)\right|\neq 0$, since $g\left(e_{i}\right)$ is an even function and has a maximum value of 0, there must exist a maximum value *K* for $g\left(e_{i}\left(t\right)\right)+h\left(e_{i}\left(t\right)\right)$ that satisfies K≥0 when |δ(t)| is bounded, which means $\dot{V}\leq K$. In this case, the error norm ${\left\|E\left(t\right)\right\|}_{F}$ may not converge to 0. However, with the increasing value of $\left|e_{i}\left(t\right)\right|$, there must exist a certain time instant *t* which satisfies $\dot{V}=0$; thus, $\left|e_{i}\left(t\right)\right|$ remains in a steady state. Therefore, there is an upper bound on the model error under bounded disturbance, and when the disturbance is 0, it is obvious that $\dot{V}\leq 0$. □

**Theorem** **2.** *The FO-ZNN will converge to the theoretical pseudoinverse globally in finite time.*

$$t=\frac{1}{\beta_{2}\left(1+r-v\right)}\ln\frac{\beta_{2}\Gamma \left(v-r+1\right){\left|e_{ij}\left(0\right)\right|}^{1+r-v}+\beta_{1}\Gamma \left(v+1\right)}{\beta_{1}\Gamma \left(v+1\right)}$$

*which is smaller than that of ZNN using FTAF*.

**Proof.**  If $e_{ij}>0$, one has

(37) $$\begin{array}{l} \frac{de_{ij}}{dt}=-\beta_{1}D^{r}e_{ij}^{v}-\beta_{2}e_{ij}\\ \;\;\;\;\;\;=-\beta_{1}\frac{\Gamma \left(v+1\right)}{\Gamma \left(v-r+1\right)}e_{ij}^{v-r}-\beta_{2}e_{ij} \end{array}$$

That means

(38) $$e_{ij}^{r-v}\frac{de_{ij}}{dt}+\beta_{2}e_{ij}^{1+r-v}+\beta_{1}\frac{\Gamma \left(v+1\right)}{\Gamma \left(v-r+1\right)}=0$$

Supposing that $z=e_{ij}^{1+r-v}$, (36) can be rewritten as

(39) $$\frac{dz}{dt}+\beta_{2}\left(1+r-v\right)z+\beta_{1}\left(1+r-v\right)\frac{\Gamma \left(v+1\right)}{\Gamma \left(v-r+1\right)}=0$$

One has the following:

(40) $$\begin{array}{l} z\left(t\right)=\left(z\left(0\right)+\frac{\beta_{1}\Gamma \left(v+1\right)}{\beta_{2}\Gamma \left(v-r+1\right)}\right)exp\left(-\beta_{2}\left(1+r-v\right)t\right)\\ \;\;\;\;\;\;\;\;-\frac{\beta_{1}\Gamma \left(v+1\right)}{\beta_{2}\Gamma \left(v-r+1\right)} \end{array}$$

Supposing that $z\left(t^{+}\right)=0$ when $t=t^{+}$, one has

(41) $$t^{+}=\frac{1}{\beta_{2}\left(1+r-v\right)}\ln\frac{\beta_{2}\Gamma \left(v-r+1\right){\left|e_{ij}\left(0\right)\right|}^{1+r-v}+\beta_{1}\Gamma \left(v+1\right)}{\beta_{1}\Gamma \left(v+1\right)}$$

Similarly, if $e_{ij}\leq 0$, one has

(42) $$t_{p}=t^{+}=t^{-}=\frac{1}{\beta_{2}\left(1+r-v\right)}\ln\frac{\beta_{2}\Gamma \left(v-r+1\right){\left|-e_{ij}\left(0\right)\right|}^{1+r-v}+\beta_{1}\Gamma \left(v+1\right)}{\beta_{1}\Gamma \left(v+1\right)}$$

According to the literature, the convergence time of the traditional dynamic equation is

(43) $$t_{t}=\frac{1}{\beta_{2}\left(1-v_{t}\right)}\ln\frac{\beta_{2}{\left|e_{ij}\left(0\right)\right|}^{1-v_{t}}+\beta_{1}}{\beta_{1}}$$

where $v_{t}\in \left(0,1\right)$. When choosing the same $\beta_{1}$ and $\beta_{2}$, suppose that $1+r-v=1-v_{t}$; that is, $v-r=v_{t}$. For $\forall v_{t}\in \left(0,1\right)$, there must exist r∈(0,1) satisfying v∈(1,2).

One has

(44) $$t_{p}-t_{t}=\frac{1}{\beta_{2}\left(1-v_{t}\right)}\ln\frac{\beta_{2}\frac{\Gamma \left(1+v_{t}\right)}{\Gamma \left(1+v\right)}{\left|e_{ij}\left(0\right)\right|}^{1-v_{t}}+\beta_{1}}{\beta_{2}{\left|e_{ij}\left(0\right)\right|}^{1-v_{t}}+\beta_{1}}$$

**Figure 3:**
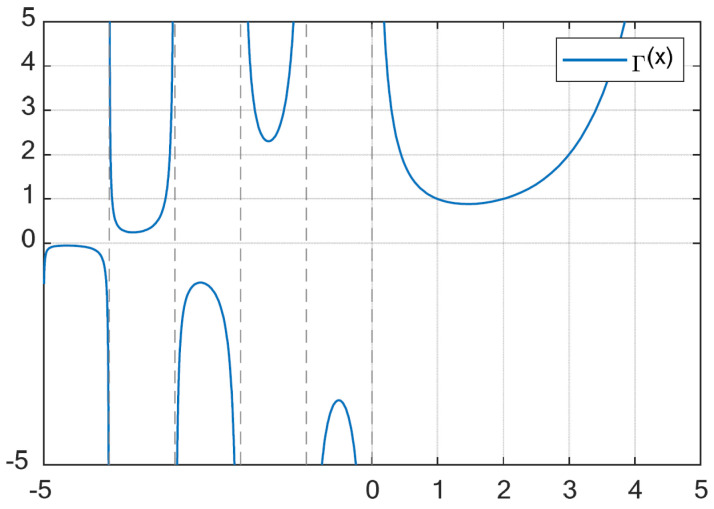
Curve of the gamma function.

As can be seen in Figure 3, the gamma function has a range of (0,1] when x∈[1,2] and a range of (1,∞) when x∈(2,∞). It can be determined that $\frac{\Gamma \left(1+v_{t}\right)}{\Gamma \left(1+v\right)}<1$. According to the properties of the gamma function, since $\frac{\Gamma \left(1+v_{t}\right)}{\Gamma \left(1+v\right)}<1$, one has

(45) $$0<\frac{\beta_{2}\frac{\Gamma \left(1+v_{t}\right)}{\Gamma \left(1+v\right)}{\left|e_{ij}\left(0\right)\right|}^{1-v_{t}}+\beta_{1}}{\beta_{2}{\left|e_{ij}\left(0\right)\right|}^{1-v_{t}}+\beta_{1}}<1$$

which means $t_{p}<t_{t}$. □

### 3.2. Design of VSMSCSG Adaptive Fractional-Order Zeroing Neural Network Steering Law

**Figure 4:**
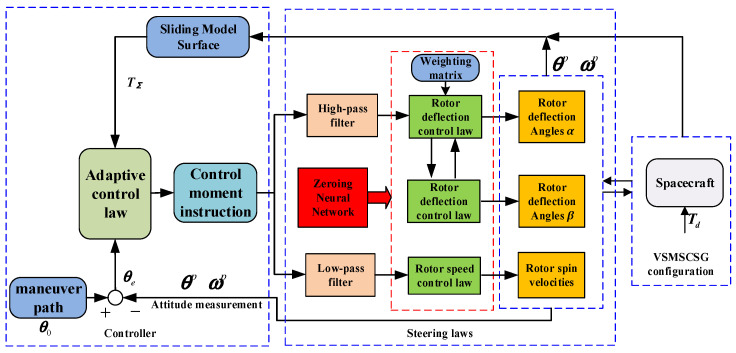
Spacecraft control system diagram of zeroing neural network steering law and adaptive control.

The control system diagram is shown in Figure 4. The control moment command given by the adaptive controller is divided into two parts by the high-pass filter and the low-pass filter. The fractional-order zeroing neural network steering law is used to derive the desired deflection angles and rotor speeds. The saturation of the rotor deflecting angles can be avoided by de-deflecting the other degrees of freedom when the angle is close to saturation. The sliding mode control is used in the attitude maneuver.

Since the rotor deflection angle of VSMSCSG is limited to ±2°, which is not conducive to the integration of attitude control and vibration suppression, a VSMSCSG adaptive control law based on deflection compensation is designed in this paper. The *X*-direction deflection is the main moment, and a nonlinear term is added to avoid rotor deflection saturation. The *Y*-direction deflection moment is the compensation moment. This moment is introduced when the deflection in the *X*-direction is saturated to compensate for the moment.

In this section, we design an adaptive fractional-order zeroing neural network steering law. First, in order to avoid saturation of the rotor deflection speed, a weighting matrix is designed as follows:

(46) $$G=\left[\begin{array}{ccc} g_{1} & 0 & 0\\ 0 & g_{2} & 0\\ 0 & 0 & g_{3} \end{array}\right]$$

where $g_{i}=1-e^{-\rho_{i}{\left|\left|\alpha_{i}\right|-\sigma \right|}^{2}}e^{-\rho_{i}{\left|\left|\beta_{i}\right|-\sigma \right|}^{2}},i=1,2,3$. σ is the extreme value of the rotor deflection angle, and $\rho_{i}$ is the weighting parameter. Thus, (4) can be rewritten as

(47) $$\dot{\alpha }=X_{A}GHu$$

(48) $$\begin{array}{l} A^{T}\left(t\right)A\left(t\right){\dot{X}}_{A}\left(t\right)=-\left({\dot{A}}^{T}\left(t\right)A\left(t\right)+A^{T}\left(t\right)\dot{A}\left(t\right)\right)X\left(t\right)\\ +{\dot{A}}^{T}\left(t\right)-\beta_{1}D^{r_{1}}{\mathrm{sgn}}^{v_{1}}\left(F\left(A^{T}\left(t\right)A\left(t\right)X\left(t\right)-A^{T}\left(t\right)\right)\right)\\ -\beta_{2}\left(F\left(A^{T}\left(t\right)A\left(t\right)X_{A}\left(t\right)-A^{T}\left(t\right)\right)\right) \end{array}$$

where ***H*** is a high-pass filter and can be expressed as

(49) $$H=\frac{s}{s+\lambda }$$

Similarly, one has

(50) $$\dot{\beta }=X_{B}\left(GHu-A\dot{\alpha }\right)$$

(51) $$\begin{array}{l} B^{T}\left(t\right)B\left(t\right){\dot{X}}_{B}\left(t\right)=-\left({\dot{B}}^{T}\left(t\right)B\left(t\right)+B^{T}\left(t\right)\dot{B}\left(t\right)\right)X\left(t\right)\\ +{\dot{B}}^{T}\left(t\right)-\beta_{1}D^{r_{2}}{\mathrm{sgn}}^{v_{2}}\left(F\left(B^{T}\left(t\right)B\left(t\right)X\left(t\right)-B^{T}\left(t\right)\right)\right)\\ -\beta_{2}\left(F\left(B^{T}\left(t\right)B\left(t\right)X_{B}\left(t\right)-B^{T}\left(t\right)\right)\right) \end{array}$$

(52) $$\dot{\Omega }=X_{c}\left(I-H\right)u$$

(53) $$\begin{array}{l} C^{T}\left(t\right)C\left(t\right){\dot{X}}_{C}\left(t\right)=-\left({\dot{C}}^{T}\left(t\right)C\left(t\right)+C^{T}\left(t\right)\dot{C}\left(t\right)\right)X\left(t\right)\\ +{\dot{C}}^{T}\left(t\right)-\beta_{1}D^{r_{3}}{\mathrm{sgn}}^{v_{3}}\left(F\left(C^{T}\left(t\right)C\left(t\right)X\left(t\right)-C^{T}\left(t\right)\right)\right)\\ -\beta_{2}\left(F\left(C^{T}\left(t\right)C\left(t\right)X_{C}\left(t\right)-C^{T}\left(t\right)\right)\right) \end{array}$$

where $\dot{\alpha }={\left[{\dot{\alpha }}_{1}\;{\dot{\alpha }}_{2}\;{\dot{\alpha }}_{3}\;{\dot{\alpha }}_{4}\right]}^{T}$, $\dot{\beta }={\left[{\dot{\beta }}_{1}\;{\dot{\beta }}_{2}\;{\dot{\beta }}_{3}\;{\dot{\beta }}_{4}\right]}^{T}$ gives the rotor deflection angular velocities, $\dot{\Omega }={\left[{\dot{\Omega }}_{1}\;{\dot{\Omega }}_{2}\;{\dot{\Omega }}_{3}\;{\dot{\Omega }}_{4}\;{\dot{\Omega }}_{5}\right]}^{T}$ gives the rotor speeds of the VSMSCSGs and the bias momentum wheel, and $u^{\prime }$ satisfies

(54) $$u^{\prime }=-{\dot{H}}_{b}$$

Equations (46)–(53) comprise the definition of the designed steering law.

## 4. Semi-Physical Simulation and Discussion

### 4.1. Comparative Simulation of Zeroing Neural Network Convergence Performance

As for the simulation conditions, the VSMSCSG system parameters obtained from experiments and the parameter settings of the controller and the steering law based on FO-ZNN are shown in Table 1. The target attitude angle is set as [0; 0; 0], and the initial attitude angle is set as [−30; 20; 40]°.

A sinusoid disturbance with an amplitude of 0.08 N·m and a frequency of 20 Hz and another one with an amplitude of 0.05 N·m and a frequency of 55 Hz are applied to the roll axis of the spacecraft.

Furthermore, a sinusoid disturbance with an amplitude of 0.015 N·m and a frequency of 60 Hz and another one with an amplitude of 0.04 N·m and a frequency of 45 Hz are applied to the pitch axis.

Finally, the yaw axis is subjected to a sinusoidal disturbance with an amplitude of 0.025 N·m and a frequency of 80 Hz and another one with an amplitude of 0.03 N·m and a frequency of 50 Hz.

The simulation comparison consists of three parts. First, we compared the convergence performance of the zeroing neural network using the proposed FOFTAF and traditional FTAF. Second, we compared the attitude control and measurement accuracy of the spacecraft controlled by the two zeroing neural network steering laws. Finally, we experimentally verified the VSMSCSG’s performance to support the above simulations.

First, the convergence accuracy and speed of zeroing neural networks using FTAF and FOFTAF as activation functions are compared through digital simulations.

**Figure 5:**
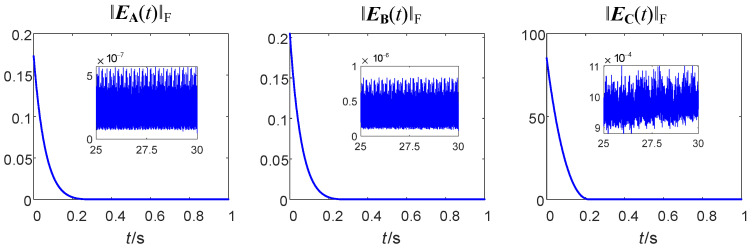
Error convergence curves using FTAF as the activation function.

Assume that ${\left\|E\left(t\right)\right\|}_{F}={\left\|e\left(t\right)\right\|}_{2}=\sqrt{\sum_{i=1}^{mn}e_{i}^{2}\left(t\right)}$ is the neural network error. The error convergence rate between the output of the zeroing neural network using FTAF as the activation function (i.e., the traditional method) and the matrix inverse is shown in Figure 5.

As can be seen in Figure 5, when FTAF is used as the activation function, the errors reach the target 0 point at 0.2 s and remain stable after that. The ranges of ‖E(t)‖ are sequentially listed as follows: 5 × 10^−7^, 7.5 × 10^−7^, 1.1 × 10^−3^.

**Figure 6:**
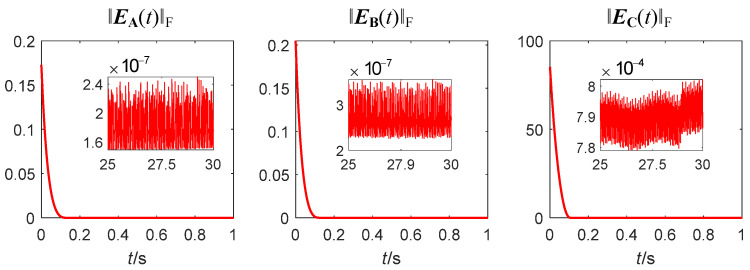
Error convergence curves using FOFTAF as the activation function.

As can be seen in Figure 6, when FOFTAF is used as the activation function, the errors reach the target 0 point at 0.1 s and remain stable after that, which is twice as fast as the FTAF method. The ranges of ‖E(t)‖ are sequentially listed as follows: 2.5 × 10^−7^, 4 × 10^−7^, 8 × 10^−4^; these ranges are reduced by 50%, 46.7%, and 27.3%, respectively, compared with those produced by the FTAF method.

Through the above simulation, it can be concluded that the convergence speed and convergence accuracy of fractional-order zeroing neural network are superior to the traditional one. This provides a basis for the performance advantages of fractional-order zeroing neural network steering law.

### 4.2. Comparison of Spacecraft Attitude Control and Measurement Accuracy and Rotor Deflection Angles Saturation

**Figure 7:**
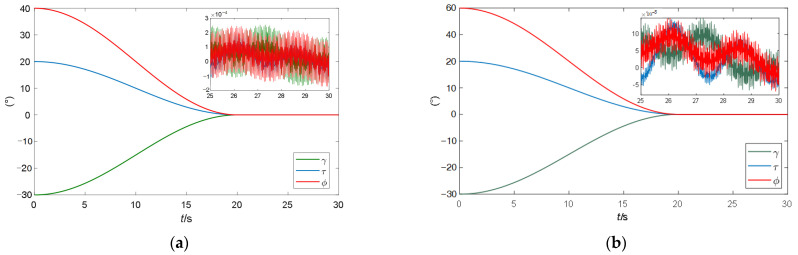
The three-axis attitude comparison curves of the spacecraft. (**a**) The spacecraft attitude controlled by the fractional-order zeroing neural network steering law; (**b**) spacecraft attitude controlled by the traditional zeroing neural network steering law.

Second, comparative simulations are carried out between the attitude control and measurement accuracy of the spacecraft controlled by the two zeroing neural network steering laws. The three-axis attitude comparison curves of the spacecraft are shown in Figure 7, in which (a) is the spacecraft attitude controlled by the fractional-order zeroing neural network steering law and (b) is the spacecraft attitude controlled by the traditional zeroing neural network steering law.

It can be seen from Figure 7 that at 20 s, the three-axis attitude angles controlled by two steering laws reach the target 0 point and remain stable after that. However, when the spacecraft attitude is stable, the fluctuation range of the three-axis attitude angles controlled by the traditional steering law is [−2 × 10^−4^°, 2.5 × 10^−4^°]. In comparison, the fluctuation range of the three-axis attitude angles controlled by the fractional-order zeroing neural network steering law is [−1 × 10^−4^°, 1.5 × 10^−4^°], which is reduced by more than 40%. This is because the error of the fractional-order zeroing neural network steering law is smaller than that of the traditional one.

**Figure 8:**
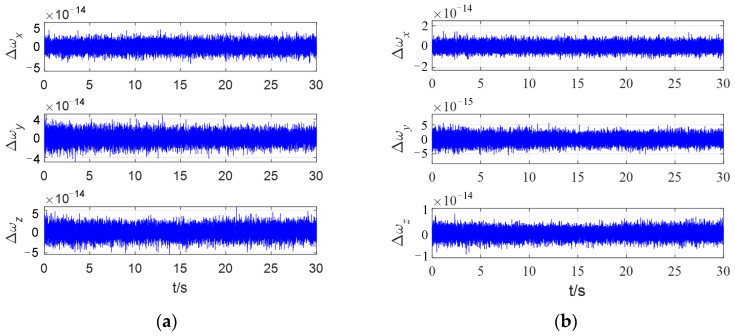
The three-axis attitude angular velocity measurement error curves of the spacecraft. (**a**) method used in [5]; (**b**) method proposed in this paper.

The attitude angular velocity measurement error is shown in Figure 8. It can be determined that after the spacecraft attitude stabilization, the three-axis measurement error ranges of the method used in [5] are sequentially listed as follows: ±3.8 × 10^−14^°/s, ±4 × 10^−14^°/s, ±5 × 10^−14^°/s; the three-axis measurement error ranges of the method proposed in this paper are sequentially listed as follows: ±1.8 × 10^−14^°/s, ±5.1 × 10^−15^°/s, ±8 × 10^−15^°/s, which are reduced by 52.6%, 87.3%, and 84%, respectively. The above results show that the proposed method improves the measurement accuracy of spacecraft attitude angular velocity.

**Figure 9:**
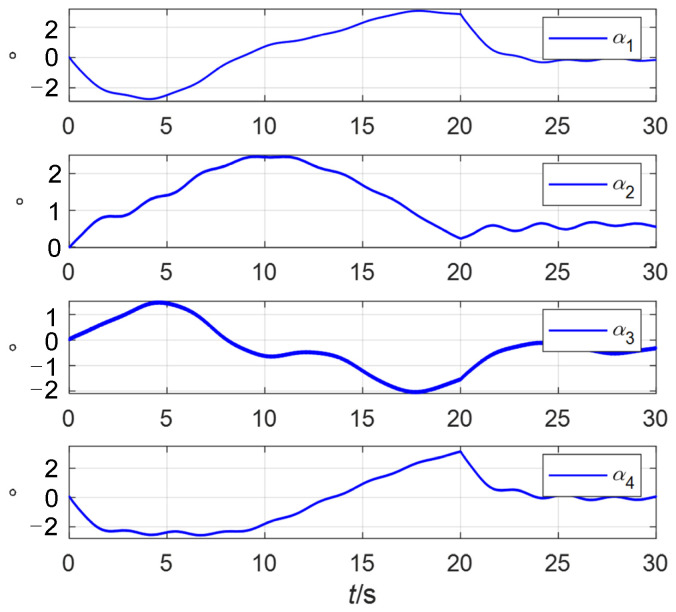
The rotor deflection angles of 4 VSMSCSGs controlled by the traditional steering law.

According to (46)–(53), the adaptive steering law proposed in this paper can avoid the saturation of the VSMSCSG rotor deflection angle. The rotor deflection angles of four VSMSCSGs controlled by the traditional steering law are shown in Figure 9.

It can be seen in Figure 9 that due to the lack of an adaptive law, when the spacecraft maneuvers, the rotor deflection angles exceed the limiting amplitude by 2°.

**Figure 10:**
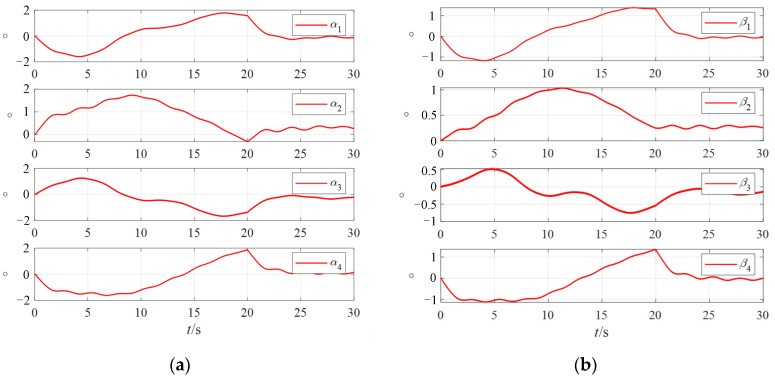
The rotor deflection angles of 4 VSMSCSGs controlled by the fractional-order zeroing neural network steering law. (**a**) Deflection angles of α; (**b**) deflection angles of β.

The rotor deflection angles of the four VSMSCSGs controlled by the fractional-order zeroing neural network steering law are shown in Figure 10.

**Figure 11:**
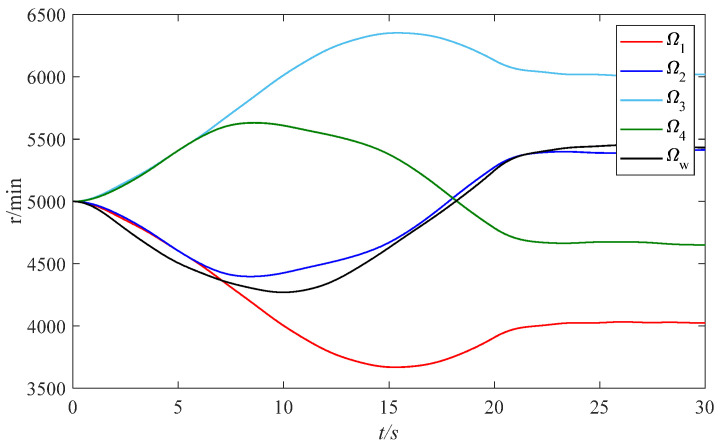
Rotor speeds of VSMSCSGs and flywheel.

It can be seen in Figure 10 that when the spacecraft maneuvers, the two-degree-of-freedom rotor deflection angles are always within the limiting amplitude. As can be seen in Figure 11, VSMSCSGs can output the attitude control moments through changing the rotor speeds, and the speeds change within a stable range.

From the above simulation, it can be determined that the proposed fractional-order zeroing neural network steering law has better performance in spacecraft attitude control and measurement and anti-saturation control of the rotors compared to the traditional steering laws.

### 4.3. High-Bandwidth Moment Output Verification Test

**Figure 12:**
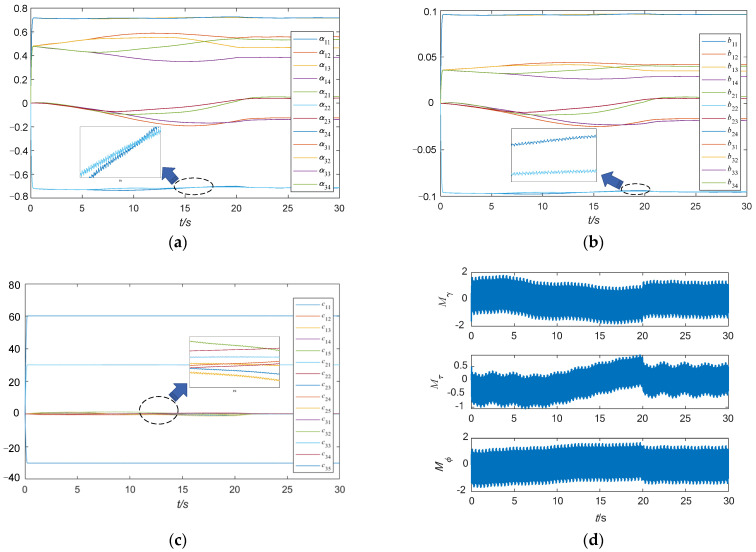
The elements of the three time-varying matrices and the control moment of the spacecraft. (**a**) The elements of ***A***; (**b**) the elements of ***B***; (**c**) the elements of ***C***; (**d**) the control moment of the spacecraft.

The elements of the three time-varying matrices ***A***, ***B*** and ***C*** and the control moment of the spacecraft are shown in Figure 12.

As can be seen in Figure 12, due to the existence of high-frequency disturbance, the bandwidth of the spacecraft’s three-axis control moment is very high, resulting in each Jacobian matrix element being a high-frequency variable, which proves that it is both feasible and necessary to use the fractional-order zeroing neural network to perform matrix inversion. Since the control moment has a high bandwidth, it is necessary to demonstrate that the VSMSCSG can output a high bandwidth control moment.

**Figure 13:**
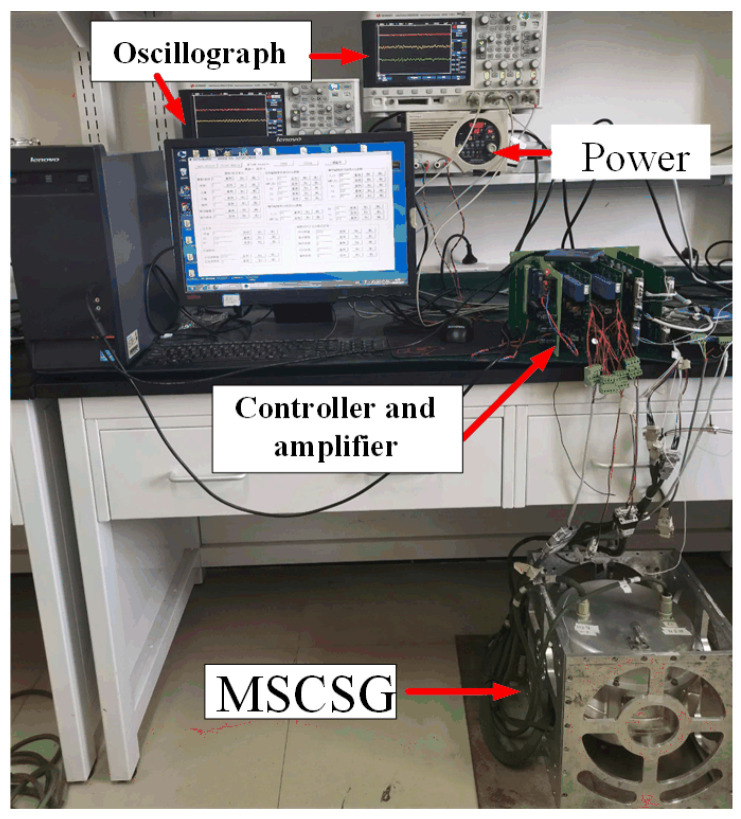
VSMSCSG laboratory setup.

In this study, a validation experiment was conducted to demonstrate that the VSMSCSG rotor is capable of outputting two degrees of freedom with a high bandwidth as the micro-frame moment and one degree of freedom with a low bandwidth as the flywheel moment. Figure 13 shows the VSMSCSG laboratory setup. The rotor deflects at a frequency of 100 Hz; meanwhile, the rotor speed changes from 4000 r/min to 3200 r/min.

**Figure 14:**
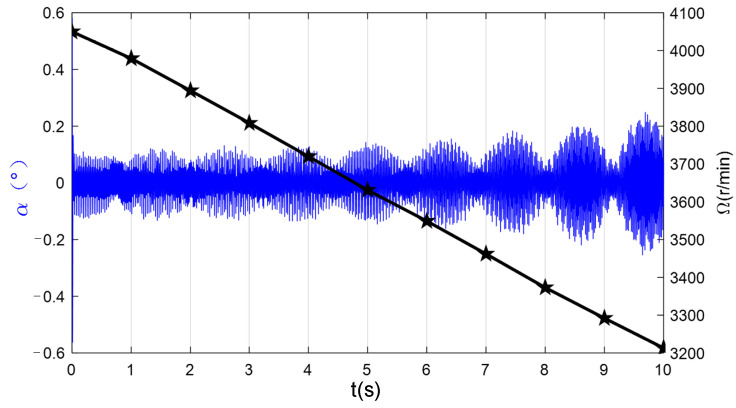
Rotor deflection angle curve and its spectrum.

Figure 14 demonstrates that the VSMSCSG rotor can deflect at a high bandwidth up to 100 Hz to output the micro-frame moment. At the same time, the rotor can change speeds to output the flywheel moment and remains stable throughout the process. These results prove the feasibility of using a VSMSCSG configuration to realize micro-vibration suppression of the spacecraft.

## 5. Conclusions

In this paper, a fractional-order zeroing neural network steering law is proposed. First, a VSMSCSG configuration is designed to realize attitude measurement and control integration. A fractional-order activation function is designed to accelerate the convergence speed of the network. Second, an adaptive zeroing neural network steering law is developed to replace the matrix pseudoinverse with the output of the FO-ZNN, and a weighting matrix with a nonlinear function is added to the steering law to ensure that the saturation and instability of the rotor spin velocity are avoided. The proposed method can be used in spacecraft attitude measurement and control integration. The simulation results demonstrate the correctness and superiority of the proposed method.

## Figures and Tables

**Table 1:** The parameter setting of the VSMSCSG system and the controller.

Parameter	Value	Parameter	Value
$J_{x}$$\;(kg\cdot m^{2}$)	0.0097	Ω (r/min)	5000
$J_{y}$$\;(kg\cdot m^{2}$)	0.0097	$f_{m}$ (Hz)	140
$J_{z}$$\;(kg\cdot m^{2}$)	0.0166	m (kg)	8.95
$\Omega_{\min}$ (r/min)	4200	λ	0.7
$\Omega_{\max}$ (r/min)	5800	$\beta_{1}$	1
$\omega_{c}$ (Hz)	0.2	$\beta_{2}$	5
*v*	1.5	*r*	0.6

## Data Availability

Data are contained within the article.
